# Oridonin Induces Apoptosis in Esophageal Squamous Cell Carcinoma by Inhibiting Cytoskeletal Protein LASP1 and PDLIM1

**DOI:** 10.3390/molecules28020805

**Published:** 2023-01-13

**Authors:** Xiaojun Zhang, Mengtao Xing, Yangcheng Ma, Zhuangli Zhang, Cuipeng Qiu, Xiao Wang, Zhihong Zhao, Zhenyu Ji, Jian-Ying Zhang

**Affiliations:** 1 Department of Biological Sciences & Border Biomedical Research Center, University of Texas at El Paso, El Paso, TX 79968, USA; 2 Henan Institute of Medical and Pharmaceutical Sciences, Zhengzhou University, Zhengzhou 450052, China

**Keywords:** oridonin, esophageal squamous cell carcinoma, proteomics, anticancer, apoptosis

## Abstract

Esophageal squamous cell carcinoma is a severe malignancy for its high mortality and poor prognosis. Mainstay chemotherapies cause serious side effects for their ways of inducing cell death. Oridonin is the main bioactive constituent from natural plants that has anticancer ability and weak side effects. The proteomics method is efficient to understand the anticancer mechanism. However, proteins identified by proteomics aimed at understanding oridonin’s anticancer mechanism is seldom overlapped by different groups. This study used proteomics based on two-dimensional electrophoresis sodium dodecyl sulfate-polyacrylamide gel electrophoresis (2-DE SDS-PAGE) integrated with mass spectrometry and Gene Set Enrichment Analysis (GSEA) to understand the anticancer mechanism of oridonin on esophageal squamous cell carcinoma (ESCC). The results showed that oridonin induced ESCC cell death via apoptosis by decreasing the protein expression of LASP1 and PDLIM1.

## 1. Introduction

Esophageal cancer is the seventh most common cancer worldwide and ranks as the sixth leading cause of cancer-related death, with 604,100 (3.1%) new cases and 544,076 (5.5%) estimated mortality as reported in 2021 [1]. The two most common histologic subtypes of esophageal cancer are esophageal squamous cell carcinoma (ESCC) and esophageal adenocarcinoma (EAC) [2]. Among esophageal cancer-causing deaths, 80% occur in ESCC [3,4]. In some areas of Asia and Africa, 90% of esophageal cancer cases are squamous cell carcinoma [5]. Esophageal carcinoma has a dire prognosis, with a 5 year survival rate of 19%, while for an advanced cancer, only 0.9% [6].

Many different approaches including surgery, chemo/radiotherapy, and endoscopic therapy are used to treat ESCC patients [3]. According to the 2019 National Comprehensive Cancer Network guidelines for esophageal cancer, the recommended therapy medicine treatments are fluorouracil/oxaliplatin, paclitaxel/cisplatin, and paclitaxel/carboplatin or cisplatin [7]. Paclitaxel, as oridonin in this study, is a diterpenoid from natural plants [8].

For the treatment of cancer or other diseases, we should understand the ways of cell death. To develop and maintain a hemostatic balance, the body is constantly generating new cells and removing unrequired or damaged cells [9]. If cell death is categorized by energy-dependent or not, apoptosis is energy-dependent which switches to independent oncosis when ATP is insufficient. If categorized by control programed or not, apoptosis is the programed cell death while necrosis, the remaining late apoptotic cells, is uncontrolled. The controlled cell deaths do not induce damage to surrounding cells and tissues, while the uncontrolled cell death spills cellular component into surrounding cells and damage them.

Most chemotherapies cause severe side effects for their ways of inducing cell death, thus, injuring the healthy cells. In this regard, it is necessary to find suitable, effective anticancer compounds with low toxicity.

**Figure 1:**
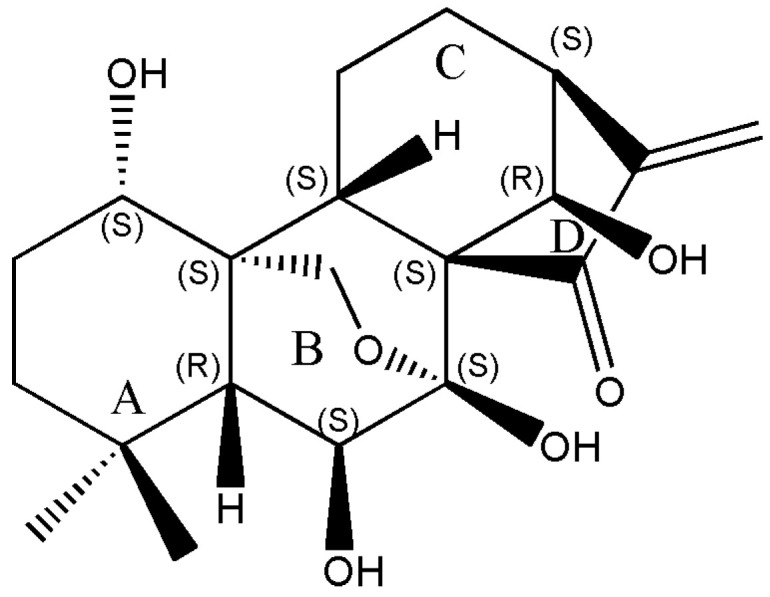
The compound structure of oridonin.

Oridonin (C_20_H_28_O_6_, MW: 364.44, Figure 1) is the main bioactive constituent of a vital traditional herbal medicine, *Rabdosia rubescens* (Hemsl.) Hara belonged to the *Isodon* genus, the *Lamiaceae* family, which has a long usage history in Asia [10]. It has been used for gastrointestinal bacterial infections and respiratory therapy as well as cancers [11]. From the mid to the end of the last century, researchers have tried to use the herb for esophageal and gastric neoplasm, and purportedly found efficacy for its entire plant [12]. As the herb’s main bioactive constituent, oridonin has anticancer efficiency and weak side effects [13].

The interactions of oridonin with its target proteins and involved signaling pathways regulate several cellular responses, including apoptosis, inflammation, autophagy, and so on [13,14]. Proteomics of two-dimensional electrophoresis sodium dodecyl sulfate-polyacrylamide gel electrophoresis (2-DE SDS-PAGE) is an effective measure to understand the anticancer bioactivity of oridonin. However, proteins identified by proteomics of different groups seldom overlapped [15,16,17,18,19,20]. In addition, two proteins, Hsp70 and Nucleolin, were identified to interact with oridonin in cancer cells. However, we still note that the anticancer bioactivity of oridonin is incompletely elucidated because these two proteins are unable to explain some of the phenomena under oridonin treatment.

In this study, 2-DE SDS-PAGE-based proteomics was used to identify differential expression proteins between oridonin-treated and untreated ESCC cells. To further understand the molecular mechanism of oridonin on ESCC cell lines, Gene Set Enrichment Analysis (GSEA) was used. The results showed that oridonin has cytotoxicity on ESCC cell lines by inducing programed cell death of apoptosis. Four proteins, LASP1, PDLIM1, ENO1, and ANXA2 involved in this process were identified through mass-spectrometry-based proteomics. Using western blotting analysis, it was shown that oridonin treatment on ESCC cell lines inhibits protein expressions of LASP1 and PDLIM1.

## 2. Results

### 2.1. Oridonin Inhibits the Proliferation and Colony Formation of Esophageal Squamous Cell Carcinoma Cells

**Figure 2:**
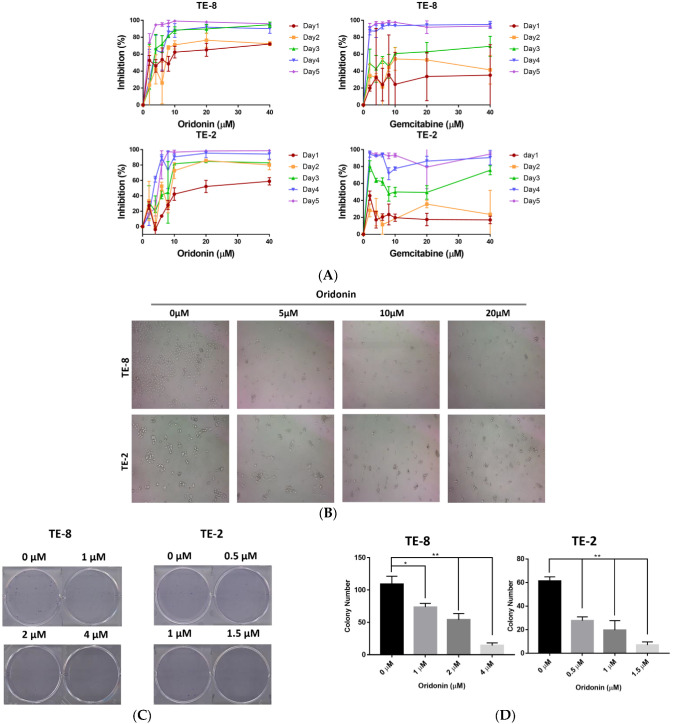
Oridonin has cytotoxicity on esophageal squamous cell carcinoma (ESCC) cells. (**A**) Oridonin inhibits the proliferation of ESCC cell lines. ESCC cells were treated with different concentrations of oridonin or Gemcitabine (positive control) for the indicated intervals. Proliferation was measured by sulforhodamine (B) (SRB) assay and inhibition% was calculated as described. IC_50_ of oridonin treatment on TE-8 for 72 h is 3.00 ± 0.46 µM, while of the positive control, Gemcitabine treatment on TE-8 for 72 h is 5.71 ± 1.07 µM. IC_50_ of oridonin treatment on TE-2 for 72 h is 6.86 ± 0.83 µM, while of Gemcitabine on TE-2 is 5.96 ± 1.11 µM. (**B**) Observation of cellular morphology upon oridonin treatment at the indicated concentrations for 72 h. Cells shrunk and rounded with increasing concentration of oridonin. (**C**,**D**) Oridonin treatment suppressed colony formation of TE-8 and TE-2 cells in a dose-dependent manner compared to the controls (* *p* < 0.05, ** *p* < 0.01). The colony number represents the cell survival ability, while the colony size shows the cell proliferation ability. Data are expressed as mean ± SEM from three independent experiments.

To evaluate the cytotoxicity of oridonin on ESCC cell lines, we first used the sulforhodamine B (SRB) assay for the proliferation and inhibition on TE-8 and TE-2 cells. Oridonin inhibits the proliferation or viability of the two cell lines in a time- and dose-dependent manner (Figure 2A). The percentage of inhibition was calculated as Inhibition% = [(OD _control_ − OD _treated_)]/OD _control_] × 100%. Representatively, the third day’s IC_50_s of oridonin on TE-8 and TE-2 are 3.00 ± 0.46 µM and 6.86 ± 0.83 µM, while Gemcitabine, anti-carcinomas chemotherapy medication [21,22], the positive control’s IC_50_s on TE-8 and TE-2 are 5.71 ± 1.07 µM and 5.96 ± 1.11 µM, respectively. Furthermore, typical apoptotic cellular morphological appearances, shrinkage, rounded, and pyknosis, were observed with increasing concentrations of oridonin treatment (Figure 2B). Moreover, oridonin reduced the number of TE-8 and TE-2 cell colonies in a dose-dependent manner (Figure 2C,D). Colony numbers of TE-8 cells treated with 1, 2, and 4 µM oridonin are 74, 54, and 14, respectively, which are significantly different from 109 for the control (* *p* < 0.05, ** *p* < 0.01). Colony numbers of TE-2 cells treated with 0.5, 1, and 1.5 µM oridonin are 28, 20, and 7, respectively, which are markedly different from 61 for the control (** *p* < 0.01).

### 2.2. Oridonin Disturbs the Cell Cycle of Esophageal Squamous Cell Carcinoma Cells

**Figure 3:**
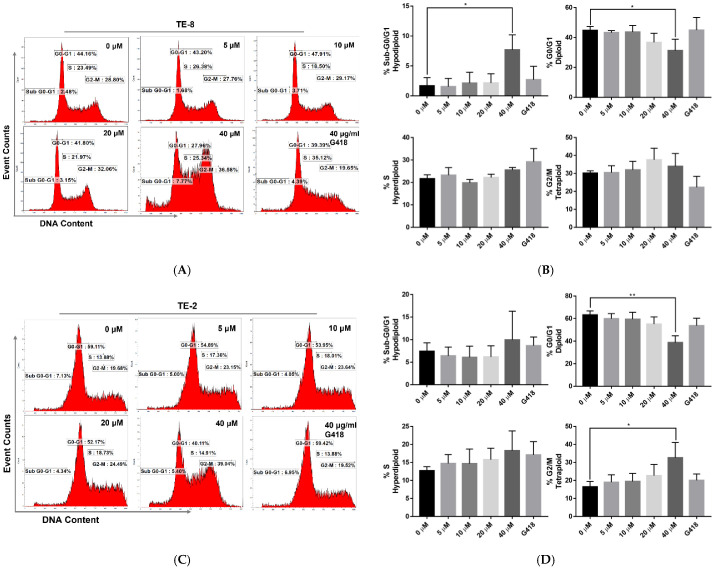
Oridonin disturbs the cell cycle of TE-8 and TE-2 ESCC cell lines. (**A**) Oridonin induces apoptotic DNA fragmentation (sub-G0/G1 phase) associated with reduced G0/G1 value in a dose-dependent manner on TE-8 cells after 24 h treatment. (**B**) Under 40 µM oridonin treatment on TE-8 cells, the sub-G0/G1 phase was significantly increased (1.68% vs. 7.68%) while the G0/G1 phase was markedly reduced (44.76% vs. 31.29%) compared to the control. (**C**) Oridonin reduced G0/G1 value and arrested the G2/M phase on TE-2 cells after 24 h treatment. (**D**) Under 40 µM oridonin treatment on TE-2 cells, the G0/G1 phase was significantly diminished (63.23% vs. 38.78%) while the G2/M phase was markedly arrested (16.43% vs. 32.60%) contrary to the control. Results are presented as mean + SEM from three independent experiments. The 0.08% dimethyl sulfoxide (DMSO) in the complete medium labeled as 0 µM was the negative control. The G418 treatment was a positive control. (* *p* < 0.05, ** *p* < 0.01).

To further confirm the cytotoxicity of oridonin on ESCC cell lines, the cell cycle distribution analysis was performed. TE-8 and TE-2 cells were treated with oridonin by 5, 10, 20, and 40 µM for 24 h, respectively. G418, a polypeptide synthesis inhibitor affecting S phase of cell cycle, was used as a positive control. For TE-8, oridonin induced DNA fragmentation and G0/G1 cell cycle reduction (Figure 3A). The sub-G0/G1 percentage was markedly higher in 40 µM oridonin-treated cells than the negative control (7.68% vs. 1.68%, *p* < 0.05, Figure 3B). As an indicator of apoptosis-induced DNA fragmentation, this sub-G0/G1 subpopulation induction was consistent with the observed morphological changes. Compared to the negative control, the G0/G1 phase from 40 µM oridonin-treated was significantly reduced (44.76% vs. 31.29%, *p* < 0.05). Though increased in a dose-dependent manner, the S and G2/M phases were not pronouncedly different. In addition, for TE-2, G0/G1 phase was reduced, and the G2/M phase was arrested by oridonin treatment (Figure 3C). The G0/G1 phase from 40 µM oridonin-treated TE-2 was significantly reduced compared to the negative control (38.78% vs. 63.23%, *p* < 0.01, Figure 3D). G2/M phase under 40 µM oridonin-treated was arrested markedly compared to the negative control (32.60% vs. 16.43%, *p* < 0.05).

Our results indicated that oridonin-treated TE-8 cells present apoptotic DNA fragmentation dose-dependently related to diminished G0/G1 values. Oridonin-treated TE-2 cells show diminished G0/G1 values and G2/M cell cycle arrest.

### 2.3. Oridonin Evokes Phosphatidylserine Externalization and Actives Caspase-3 on Esophageal Squamous Cell Carcinoma Cells

**Figure 4:**
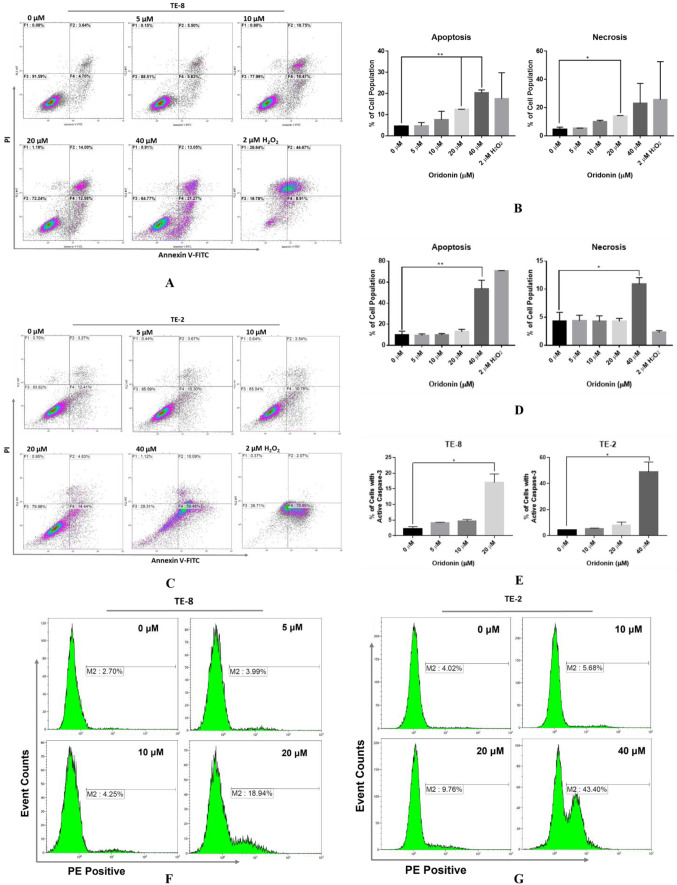
Oridonin induces phosphatidylserine externalization and caspase-3 activation on TE-8 and TE-2 cell lines. (**A**) Oridonin leads to TE-8 cells dose-dependently accumulated in early- and late-stage/necrosis apoptosis with treatment for 24 h. (**B**) The percentages of early apoptosis treated with 20 and 40 µM oridonin and late-stage/necrosis apoptosis treated with 20 µM oridonin on TE-8 were significantly different from the negative control. (**C**) Oridonin causes apoptosis of TE-2 cells after 48 h treatment although concentration-dependent was not observed. (**D**) The percentage of early-apoptosis upon 40 µM oridonin treatment on TE-2 was markedly different to the negative control. (**E**) Compared to the negative control, oridonin treatment under 20 and 40 µM on TE-8 or TE-2 cell lines for 48 h showed significantly activated caspase-3. (**F**) 20 µM oridonin induces caspase-3 activation on the TE-8 cell line. (**G**) 40 µM oridonin induces caspase-3 activation on the TE-2 cell line. Results are presented as mean + SEM from two independent experiments. The H_2_O_2_ treatment was a positive control. The 0.08% DMSO in the complete medium labeled as 0 µM was the negative control. (* *p* < 0.05, ** *p* < 0.01).

TE-8 and TE-2 cells were treated with oridonin at 5, 10, 20, and 40 µM followed by Annexin V-FITC/PI staining and flow cytometer to illustrate the apoptosis inducing potential. The 2 µM hydrogen peroxide (H_2_O_2_) was a positive control while 0.08% DMSO in the complete medium was the negative control. Oridonin leads to TE-8 cells dose-dependently accumulated in early- (Annexin V+/PI−) and late-stage/necrosis (Annexin V+/PI+) (Figure 4A). The percentages of early-stage (Annexin V+/PI−) apoptosis under 20 and 40 µM oridonin treatment (12.5% and 20.3%) were significantly higher than the negative control (4.6%, *p* < 0.01, Figure 4B). The percentage of late-stage/necrosis (Annexin V+/PI+) apoptosis under 20 µM oridonin treatment (14.0%) was pronouncedly different to the negative control (4.7%, *p* < 0.05, Figure 4B). For TE-2 cell line, the percentage of early-(Annexin V+/PI−) apoptosis under 40 µM oridonin was 53.72% compared to 10.12% for the negative control (*p* < 0.01) while the late-stage apoptosis was 10.91% versus 4.34%, which were markedly different, though concentration-dependent was not observed (*p* < 0.05, Figure 4C,D).

As the convergence point of both upstream intrinsic and extrinsic apoptotic pathways, activation of caspase-3 was tested. TE-8 or TE-2 cells exposed to oridonin upon 20 and 40 µM exhibited significantly activated caspase-3 after 48 h treatment compared to the negative control (17.05% vs. 2.18%, 49.01% vs. 4.21%, *p* < 0.05, Figure 4E–G). After oridonin exposure from 5 µM to 20 µM, the percentage of TE-8’s caspase-3 activation increased ~4 fold while increased ~9 fold in TE-2 after exposure from 10 µM to 40 µM.

### 2.4. Two-Dimensional Electrophoresis-Based Proteomics Identifies Proteins That Are Impacted by Oridonin

**Figure 5:**
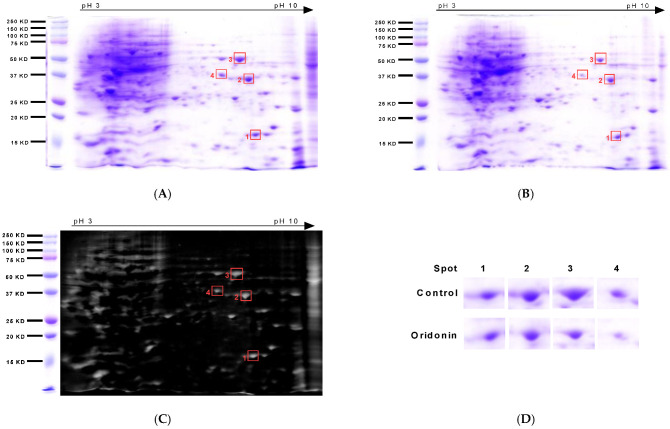
Different expression of proteins that are influenced by oridonin treatment identified by proteomics. (**A**) The global view of proteins of untreated TE-8 cells. (**B**) The global view of proteins of 40 µM oridonin-treated TE-8 cells for 24 h. (**C**) Different expression of proteins of oridonin-treated to untreated TE-8 cell lysates. Cell lysates of same amount proteins were first separated by 7 cm IPG strips and then the SDS-PAGE gels for the second dimensional isolation. Finally, the gels were stained with Coomassie blue. Different expression of protein image was reached by the MATLAB image processing module. (**D**) Close-up spot images of pronounced changed protein spots.

The proteome analysis approach was used to identify target proteins influenced by oridonin for anticancer ability. Combined with genomics advancement and mass spectrometry, 2-DE SDS-PAGE has permitted the simplistic identification of target proteins. Loading the same quantitative proteins (Table S1), the global views of TE-8 cells treated and untreated by oridonin were obtained by 2-DE SDS-PAGE using a wide pH range of 3–10 non-linear IPG strips for IEF followed by the SDS-PAGE with Coomassie blue staining (Figure 5A,B). The different expression of protein spots of treated to untreated cell lysates were gained by the MATLAB imaging processing module (Figure 5C). Four close-ups of pronounced changed protein spots were shown (Figure 5D). These protein spots were excised from the gel, digested, and extracted, allowing protein identification using mass-spectrometry-based techniques.

Eight putative proteins were identified, whose gene names were *UBE2V2, UBE2V1, ANXA2, VDAC2, ENO1, PDLIM1, LASP1,* and *HNRPH3* (Table 1). Next step, we run a bioinformatics analysis screening out more related protein.

### 2.5. Oridonin Treatment Inhibits LASP1 and PDLIM1 Expression on ESCC

Eight potential proteins involved in the process of oridonin anticancer activity on ESCC were identified. The observed phenomena including morphological changes and flow cytometry analysis are related to the apoptosis. Whether the potential proteins are consistent with the observed apoptotic presentation? Which one is the key protein for the next step? What is the relationship between proteins identified by the current study and the known proteins identified by proteomics of other groups? Searching google scholar by keywords of proteomics and oridonin, six articles were found [15,16,17,18,19,20]. To resolve these questions, a pool of genes encoding the proteins identified by proteomics from them including the current study was made (Table S2). The GSEA analyzed the molecular function, cellular component, and biological process of these genes.

**Figure 6:**
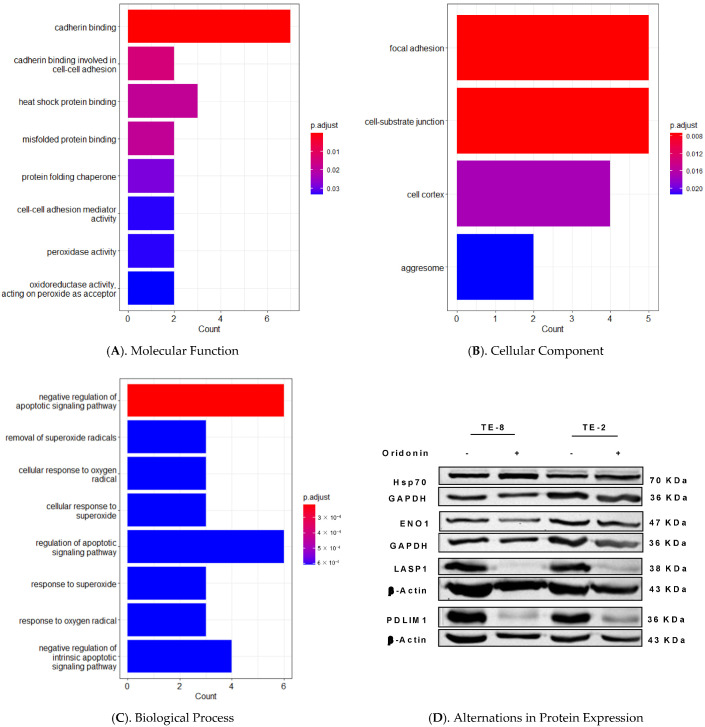
Gene Set Enrichment Analysis and western blotting assessed that oridonin inhibits LASP1 and PDLIM1 on ESCC. (**A**) Molecular function classification of the protein candidates involved in oridonin treatment. The top three are cadherin binding, cadherin binding involved in cell–cell adhesion, and heat shock protein binding. (**B**) The top three cellular component classifications are focal adhesion, cell-substrate junction, and cell cortex. (**C**) The top three biological processes are negative regulation of apoptotic signaling pathway, removal of superoxide radicals, and cellular response to oxygen radicals. (**D**) Protein expression of LASP1 and PDLIM1 under oridonin treatment decreased compared to untreated cell lysates. Hsp70 expression increased in oridonin-treated cell lysates compared to untreated while ENO1 decreased a little in treated TE-8.

The candidates were classified into three groups based on their Gene Ontology (Figure 6A–C). The top three significantly related molecular functions are cadherin binding, cadherin binding involved in cell–cell adhesion, and heat shock protein binding (Figure 6A). The top three markedly reported cellular components are focal adhesion, cell-substrate junction, and cell cortex (Figure 6B). In addition, the top three ranked pronouncedly related biological processes are the negative regulation of apoptotic signaling pathway, removal of superoxide radicals, and cellular response to oxygen radicals. The results are interesting because they are consistent with the cytotoxicity and flow cytometry results, including the morphological changes of apoptosis in this study.

The interested genes were listed according to the significantly ranked Gene Ontology terms by their hit times (Table 2, Figure S1). The gene list includes six genes: *HSPA1A, HSPA1B, LASP1, PDLIM1, ENO1*, and *ANXA2*. The list also shows the hit times of each gene, except that already known Hsp70, *LASP1* and *PDLIM1* are four times while *ENO1* and *ANXA2* are three times. Their protein expressions were assessed by western blotting (Figure 6D, Table S3). As reported, Hsp70 increased in the oridonin-treated cell lysates [15,16]. Compared to the untreated, oridonin-treated cell lysates decreased the protein expression of LASP1 and PDLIM1 in both TE-8 and TE-2 cell lines. The expression of ENO1 slightly decreased in TE-8.

## 3. Conclusions and Discussion

Our main finding is that oridonin induces apoptosis on ESCC cells (TE-8 and TE-2) by impacting protein expression of LASP1, PDLIM1, ENO1, and ANXA2. The cytotoxicity of oridonin on ESCC cells was evidenced by inhibiting the cell proliferation and colony formation. Oridonin treatment produced apoptotic DNA fragments on TE-8 cells by increasing the sub-G0/G1 phase and reducing the G0/G1 phase under 40 µM treatment. Oridonin reduced the G0/G1 phase of TE-2 cells and arrested the cell cycle at the G2/M phase upon 40 µM treatment. The apoptotic phenomenon was further proved by the Annexin V-FITC/PI double staining flow cytometry. The activated caspase-3 also showed the involvement of the caspase cascade pathway. In order to know the proteins/pathways influenced by oridonin treatment, we used mass-spectrometry-based 2-DE SDS-PAGE gel. Identification of excised protein spots with GSEA and western blotting guided us to several proteins: LASP1, PDLIM1, ENO1, Hsp70, and ANXA2.

This study focuses on the proteome rather than the transcriptome and genome. At the RNA and DNA levels, researchers have already achieved remarkable results. As confirmed through transcriptomics in ESCC, glutathione depletion is involved in oridonin cytotoxicity. Oridonin increased the GSH-ROS controlling genes and ROS production while decreasing the intracellular GSH [23]. On t(8;21) acute leukemia myeloid, oridonin induced apoptosis and targets the important AML1-ETO fusion protein in leukemogenesis [24]. Oridonin also acts with several critical signaling pathways such as AKT [25], Ras/Raf [26], JNK [27], and NLRP3 [28]. At present, using proteomics including 2D SDS-PAGE and chemical proteomics, six articles pointed out 24 impacted proteins that were conducted as the gene pool in the current study [15,16,17,18,19,20]. Among them, Hsp70 1A and Nucleolin were proved to be the targets of oridonin [15,16,17]. Maybe for the different cell lines and mechanisms, proteins found by proteomics from different groups are very different and cannot explain all. This study not only identified influenced proteins of oridonin in ESCC anticancer mechanism, but also tried to see the relationship of proteins identified by proteomics till now. 

However, this study has limitations. First, we only identified several expression spots without all differentiation spots. Though LASP1 and PDLIM1 are affected by oridonin, they may be downstream of the actual targets of the compound. However, the KEGG pathway analysis shows that LASP1 and PDLIM1 are not involved in the five significantly oridonin-related pathways: prion disease, spliceosome, one carbon pool by folate, antifolate resistance and ferroptosis (Figure S2 and Table S4). Oridonin’s function of ferroptosis on TE1 cells has been reported [29]. However, this does not mean that LASP1 and PDLIM1 are not important. It is simply evidence of the novelty of these two proteins due to the less-related proteins on related pathways in the gene pool that have been reported up until now. Secondly, the 2D SDS-PAGE method has defection. Except for the high technique and complicated requirement, the target of the compound could be a low abundant protein or out of the detected limitation of this method. Moreover, a gene pool from all known influenced proteins, RNA, and DNA, is expected. Finally, the label-free quantification using 2D SDS-PAGE has space to be improved.

For the cell cycle analysis, oridonin-treated ESCC at 5, 10, 20, and 40 µM for 24 h and the calculated IC_50s_ (both at 24 and 72 h) were much lower. One possible reason is that oridonin impacts cells’ attaching ability. For the proliferation experiment, cells were stained and suffered several times of washing. The weakly attached cells were removed, which influences the IC_50_ while the cell cycle collected both floating and attaching cells. Another possible reason is that oridonin has different cytotoxicity according to the cell density. The seeding cell density of the cell cycle was around two times that for cell proliferation, 6 × 10^5^/21 cm^2^ to 5000 cells/0.32 cm^2^.

Lim, actin, and SH3 domain protein (LASP1) has a distinctive domain configuration containing SH3 and LIM. It has an N-terminal cysteine-rich LIM domain and carboxyl-terminal *src* homology (SH3) domain without nebulin and nebulette. The F-actin binding domains with nebulin-like repeats (NR) are localized at the middle region next to the LIM domain. It was initially identified as a structural cytoskeletal protein for actin-binding and is overexpressed 8–12% in breast cancer patients and other cancers [30]. After, it was developed as a cancer prognostic biomarker for many cancers and a nuclear transcriptional regulator [31,32]. This protein has been reported to play roles in tumor invasion, metastasis, and epithelial to mesenchymal transition (EMT).

For migratory cells, LASP1 is spatially regulated and a dynamic focal adhesion protein. Rather than the central body of the migrating cells, LASP1 is strongly polarized and mainly located at the leading edge of pseudopodium and focal adhesions podosomes. The COOH-terminal portion, the SH3 domain, plays a critical role in the response of targeting to focal adhesions. Due to apoptotic stimulation, activated c-Abl, the cytoskeletal regulatory protein, selectively phosphorylates tyrosine 171 of LASP1, which prevents LASP1’s translocation into focal complexes [33]. However, instead of migrating cells, c-Abl only impacts apoptotic cells’ focal adhesion. Cytochrome *c* releasing is also considered to be involved in Abl-mediated cell death. An early event of apoptosis includes the focal adhesion remodeling and detachment of cells from the extracellular matrix. The rounded morphological change of ESCC cells under oridonin treatment is consistent with the loss attachment phenomenon of apoptosis.

PDLIM1, alternatively called CLP36, Elfin, or CLIM1, is a cytoskeleton regulation protein as one member of the PDL-LIM family by binding with α-actinin [34]. This protein has a PDZ domain at the amino terminus, while at the carboxyl terminus is a LIM domain. PDLIM1 regulates the actin cytoskeleton organization and is involved in the cells’ morphological changes and migration [35]. The expression level of PDLIM1 impacts the assembly of focal adhesion. Different from NR of LASP1, which is responsible for F-actin binding, the LIM domain of PDLIM1 is in charge of binding to actin cytoskeletal components.

ENO1 has been previously identified as a tumor prognostic biomarker and its expression impacts cancer cells’ proliferation, migration, and invasion [36,37,38]. It is a potential target of immunotherapies [39]. In addition, it might be a promising target for the treatment and a prognostic marker for human gastric cancer [40]. The location of ENO1 is mainly at the cytoplasm while the nuclear form is an alternative one. Enolase (ENO1) is a glycolytic enzyme involved in glucose metabolism and tumor progression [41]. It participates in the synthesis of pyruvate in aerobic glycolysis in cancer cells. In lung cancer cells, a reduced expression of ENO1 attenuated glycolysis through ENO1 by reducing the ATP level and retarding the relative glucose uptake, finally inducing apoptosis [41].

Being a member of the annexin family such as Annexin V (ANXA5), ANXA2 plays a key role in apoptosis [42]. This calcium-mediated phospholipid-binding protein family consists of membrane-binding proteins. It is unsurprising that cells induced by oridonin changed protein expression of ANXA2 when they had already been verified of apoptosis via ANXA5. This is explicit evidence of echoing among the projects.

In summary, oridonin has cytotoxicity on ESCC cells through induced cell death via apoptosis by decreasing cytoskeletal protein LASP1 and PDLIM1, while increasing Hsp70 expression and slightly inhibition of ENO1.

## 4. Materials and Methods

### 4.1. Cell Lines and Cell Culture

TE-8 and TE-2 cell lines, 2 human ESCC cell lines, were donated by a collaborator at the University of Illinois at Chicago [43,44]. TE-8 is a medium while TE-2 is a poorly differentiated squamous cell line. Cells were fostered with complete RPMI 1640 medium (Hyclone, GE Healthcare Life Sciences, Logan, UT, USA) by 10% fetal bovine serum addition (Corning, Mediatech, Manassas, VA, USA). The regular water-jacketed cell incubator was set at 37 °C with 5% humidified CO_2_.

### 4.2. Cell Proliferation and Inhibition Assay

Oridonin compound was purchased from Beijing OKA Biological Technology Co., Ltd. (Beijing, China), and the purity was confirmed to be ≥98%. The oridonin solution was prepared in DMSO (Sigma, MO, USA). Gemcitabine hydrochloride was purchased from Selleck Chemical (Houston, TX, USA) and made up in sterile phosphate-buffered saline (PBS) with the stock solution of 160 mg/kg.

Seed cells on 96-well plates by 5000 cells/well and treat with Oridonin, with, as indicated, final concentrations for a different duration. Fix cells in situ with 40% (*w/v*) cold Trichloroacetic acid (TCA). Let them sit for 5 min. Incubate plates at 4 °C for 1 h. Discard supernatant and gently wash plates 5 times with water, followed by an air-dry. Stain fixed cells with 0.4% (*w/v*) SRB with 1% acetic acid as solubilizer. After staining, wash with 1% acetic acid to remove the free dye and air-dry. Solubilize the bound stain with 100 mM Tris Base for reading. Read absorbance on a microplate reader at 515 nm [45,46]. Inhibition% = [(OD _control_ − OD _treated_)]/OD _control_] × 100%.

### 4.3. Colony Formation Assay and Cellular Morphological Changes

Seed single-cell suspensions in 6-well plates by 500 cells/well, in triplicate. After attached, treat cells with the indicated concentration of Oridonin for 72 h, and then change with fresh complete medium, and incubate for 5 days. Observe cells by microscope. Rinse colonies with PBS, fix with methanol at room temperature for 15 min, and finally stain with 0.1% crystal violet for 20 min. Count the clearly visible colonies (foci > 50 cells) after washing [47,48].

### 4.4. Cell Cycle Profile Analysis

Seed cells on 60 mm Petri dishes at 6 × 10^5^/dish density. After attachment, treat cells with the indicated concentrations of oridonin for 24 h and then harvest. Collect both floating and adherent cells and fix in 70% ethanol at 4 °C overnight [47,49]. Then, incubate the fixed cells with RNase at 50 µg/mL at 37 °C for 5 min and stain with Propidium Iodide (PI) solution 5 µL/mL (MP Biomedicals, Solon, OH, USA) and immediately process via flow cytometry. G418 was the positive control while 0.08% DMSO in the complete medium was the negative control. By gating cells of the flow cytometer (Gallios; Beckman Coulter, Miami, FL, USA), acquire around 20,000 events in each sample. Use Kaluza software (Beckman Coulter, Miami, FL, USA) for the cell cycle phase distribution: sub-G0/G1 for apoptosis-induced DNA fragmentation indication as hypodiploid; G0/G1 for diploid; hyperdiploid as S; and tetraploid as G2/M. For each experimental purpose and their associated controls, three replicas were performed.

### 4.5. Analysis of the Phosphatidylserine Distribution via Annexin V-FITC/PI Assay

Seed cells at a density of 6 × 10^5^/dish on 60 mm Petri dishes. After attachment overnight, treat cells with indicated concentrations oridonin for 24 h or 48 h. Collect and wash both floating and trypsinized cells with cold PBS [50,51]. Detect apoptosis with Annexin V-FITC/PI apoptosis kit (BD Pharmingen, San Jose, CA, USA). As per the manufacturer’s instructions, reagents are added and incubated. Finally, analyze the cell apoptosis by a flow cytometer (Gallios; Beckman Coulter, Miami, FL, USA). The apoptotic cells mean the early apoptosis (Annexin V-FITC-positive) and late/necrotic apoptotic (Annexin V-FITC-positive, PI-positive) cells.

### 4.6. Detection of Caspase-3 Activation

Seed cells at a density of 6 × 10^5^/dish on 60 mm Petri dishes and allow attachment overnight. Then, treat with oridonin for 48 h at indicated concentrations. Detect PE Active caspase-3 by following the manufacturer’s introductions (BD Pharmingen, San Jose, CA, USA). Briefly, resuspend the cell pellets in Cytofix/Cytoperm solution at 1 × 10^6^ cells/0.5 mL. Incubate cells on ice for 20 min. Centrifuge and wash cell pellets twice with Perm/Wash buffer at 0.5 mL buffer/1 × 10^6^ cells. Resuspend cells in Perm/Wash buffer with caspase antibody and incubate for 30 min. At the end of incubation, analyze the PE active caspase-3 on a flow cytometer (Gallios; Beckman Coulter, Miami, FL, USA). Quantify the non-apoptotic (M1) and apoptotic (M2) populations.

### 4.7. 2-Dimensional Electrophoresis SDS-PAGE Gel Analysis

Lyse the cells treated by oridonin at 40 µM for 24 h or untreated in 2-dimensional electrophoresis (2-DE) rehydration buffer (Bio-Rad, Hercules, CA, USA) and vigorously vortex them for 90 min, then ultrasonicate [52]. Remove insoluble substances by centrifuge and collect the supernatant for protein quantification by the Bradford Protein Assay (Bio-Rad, Hercules, CA, USA). For isoelectric focusing (IEF) analysis, apply the same quantitative proteins in 125 μL rehydration buffer on a pH 3–10, 7 cm IEF strip (Bio-Rad, Hercules, CA, USA). Perform IEF at 50 mA/gel, 250 V for 30 min, followed by a linear increase to 4000 V in 90 min, and maintained at 4000 V for 25,000 Vhour [53]. Use 12% of sodium dodecyl sulfate-polyacrylamide gels (SDS-PAGEs) for the second-dimensional electrophoresis. Stain proteins with 0.1% Coomassie blue R-250. Analyze the different expression of protein spots of treated to untreated cell lysates by MATLAB imaging processing module after transferring to the rgb2gray function.

### 4.8. Sample In-GEL Trypsin Digestion for Mass Spectrometry

Excise differentially expressed protein spots from Coomassie blue-stained gels and expose them to an In-Gel Tryptic Digestion Kit (ThermoFisher, Waltham, MA, USA) as described by the manufacturer. Briefly, subject excised samples to 200 µL Destaining Solution and incubated at 37 °C for 30 min with constant shaking. Reduce samples with 50 mM TCEP (Thermo Scientific, Waltham, MA, USA) and allow to incubate for 10 min at 60 °C, followed by alkylation with 106 mM Iodoacetamide (IAA) for 1 h in the dark, at room temperature. Shrink the gel piece with OPTIMA grade acetonitrile for 15 min. Discard the acetonitrile, and allow the gel piece to dry completely. Digest the prepared proteins with 10 ng/µL Trypsin (Sigma, St. Louis, MO, USA) into a 25 µL Digest Solution. Digest samples at 30 °C for 12 h with constant shaking. The remove the remaining supernatant (35 µL) and place at −20 °C until liquid chromatography-tandem mass spectrometry (LC-MS/MS) acquisition.

### 4.9. Liquid Chromatography High-Resolution Mass Spectrometry

Load peptides onto a Nano-LC C18 analytical column (µPAC, 200 cm; PharmaFluidics, Ghent, Belgium) with 4% solvent B (100% acetonitrile, 0.1% formic acid) and 96% solvent A (100% water, 0.1% formic acid) at a flow rate of 0.5 µL/min for 10 min. Separate peptides with a Dionex Ultimate 3000 RSLCnano (Thermo Scientific, Waltham, MA, USA) at 10.1 min with the flow rate decreased to 0.3 µL/min and increased to 22% solvent B over 129.9 min. Increase Solvent B to 45% over 50 min and to 95% over 10 min. Maintain a high organic plateau of 95% solvent B for 19 min before decreasing to 4% over 1 min. Re-equilibrated the column with 4% solvent B for 30 min before the next sample injection for a total of 250 min runtime. Ionize the eluted peptides with a µPAC Flex iON Connect (PharmaFluidics, Ghent, Belgium) with a nanoESI emitter (FOSSILIONTECH, Madrid, Spain) attached to a Nanospray Flex ion Source (Thermo Scientific, Waltham, MA, USA). Analyze the ionized peptides with a Q-Exactive Plus Hybrid Quadrupole-Orbitrap mass spectrometer (MS; ThermoFisher Scientific, Waltham, MA, USA) with MS1 resolution at 70,000, scan range from 350 to 1400 *m/z*, and an AGC target at 1 × 10^6^. Set the data-dependent MS2 parameters to 17,500 resolution, AGC target 1 × 10^5^, nce 27. Set the charge exclusion ions to unassigned, +1, +6–8, >8.

### 4.10. Mass Spectrometry Analysis of Different Expression of Proteins

Database searching: All MS/MS samples were analyzed using Sequest (version IseNode in Proteome Discoverer 2.5.0.400; Thermo Fisher Scientific, Waltham, MA, USA). Sequest was set up to search Human_Database_043021.fasta (171,131 entries), assuming the digestion enzyme was trypsin. The fragment ion mass tolerance of 0.020 Da and precursor ion tolerance of 10.0 PPM were set for searching. The carbamidomethyl of cysteine was set as a fixed specified modification. Met-loss of methionine, met-loss + Acetyl of methionine, oxidation of methionine, and acetyl of the N-terminus were specified in Sequest as variable modifications.

Criteria for protein identification: MS/MS-based peptide and protein identifications were validated by Scaffold (Proteome Software, Inc., version 5.0.1). Peptide identification thresholds were set at ≥95.0% probability of the Peptide algorithm [54]. Protein identification thresholds were set at ≥99.0% probability and included at least 2 identified peptides. The Protein Prophet algorithm was used for protein probabilities assignment [55]. Mass spectrometry queries were performed by using both the Scaffold software on the database of NCBI (National Center for Biotechnology Information) and Uniprot.

### 4.11. Gene Set Enrichment Analysis

R software (version 4.1.2) was used to read and analyze genes encoding the proteins identified by proteomics for their Gene Ontology (GO) annotations. The Bioconductor package “clusterProfiler” (version 4.2.1) was used to perform GO annotations and probe ID was annotated to gene ID through “org.Hs.eg.db” database [56,57]. The *p*-value of the analysis was set at 0.05.

### 4.12. Western Blotting Analysis

Cells were cultured and treated with 30 µM oridonin for 48 h. Cell lysates were obtained using lysis buffer (2% Tris-HCl (1 M, pH7.4), 15% Glycerol, 1% Triton X-100, 8 mM MgSO_4_, 150 mM NaCl, 1 mM EDTA) with fresh Protease and Phosphatase Inhibitor (Thermo Scientific, Cat# P17443, Waltham, MA, USA). Total protein lysates were quantified by the Bradford Protein Assay (Bio-Rad, Cat# 500-0201, Hercules, CA, USA) and separated on SDS-PAGE, followed by transfer to nitrocellulose membranes (GE Healthcare Life Sciences, Pittsburgh, PA, USA). Membranes were blocked with 5% Beef Serum Albumin (BSA) and then incubated with primary antibodies: mouse Hsp70 antibody (mAb; Abcam, Boston, MA, USA), rabbit ENO1, LASP1, GAPDH antibodies (#3810 (pAb), #8636 (pAb), #2218 (mAb); cell signaling, Danvers, MA, USA), rabbit PDLIM1 antibody (ABclonal, Cambridge, MA, USA), and mouse β-Actin antibody (Santa Cruz Biotechnology Inc., Santa Cruz, CA, USA). Then, they were incubated with horseradish peroxidase (HRP)-conjugated secondary antibody to rabbit (BD Pharmingen, San Jose, CA, USA) and HRP-conjugated IgG to mouse (BD Pharmingen, San Jose, CA, USA). Enhanced chemiluminescence substrate (Thermo Scientific, Waltham, MA, USA) was used for development. The images were captured by the iBright FL1000 imaging system (Invitrogen, Waltham, MA, USA).

## Figures and Tables

**Table 1:** Top Candidate Proteins Involved in Oridonin-treated ESCC.

Spot No.	Protein Description	MW ^a^ (KDa)	pI ^b^	No. of Total Spectra	Coverage (%)	Exclusive Peptides ^c^	MS ^d^ Scores (%)	Density Decrease Ratio (%)
1 ^e^	Ubiquitin-conjugating enzyme E2 variant 2	16	7.79	57	62	4	100	13.1
	Ubiquitin-conjugating enzyme E2 variant 1	16	7.71	50	57	1	100	
2 ^e^	Annexin A2	39	7.57	23	46	14	100	37.3
	Outer mitochondrial membrane protein porin 2	34	7.50	20	35	9	100	
3	Alpha-enolase	47	7.01	2134	76	19	100	60.1
4 ^f^	Epididymis secretory protein Li 112	36	6.56	86	31	10	100	63.9
	LIM and SH3 domain protein 1	30	6.61	24	39	9	100	
	Heterogeneous nuclear ribonucleoprotein H3	37	6.37	4	10	3	99	

^a^. Molecular weight, ^b^. Isoelectric point, ^c^. Exclusive unique peptides or exclusively percentage, ^d^. Mass spectrometry, ^e^. Two potential proteins from a single spot, ^f^. Three potential proteins from a single spot.

**Table 2:** Interested Genes for Protein Assessment Involved in Oridonin Treatment.

		HSPA1A (5)	HSPA1B (4)	LASP1 (4)	PDLIM1 (4)	ENO1 (3)	ANXA2 (3)
Molecular Function	Cadherin binding	X		X	X	X	X
	Cadherin binding involved cell-cell adhesion				X		X
	Heat shock protein binding	X	X				
Cellular Content	Focal adhesion	X	X	X	X		
	Cell substrate junction	X	X	X	X		
	Cell cortex			X		X	X
Biological Process	Negative regulation of apoptotic signaling pathway	X	X			X	
	Removal of superoxide radicals						
	Cellular response to oxygen radical						

## Data Availability

Data supporting this reported study can be found within the maintext and Supplemental Materials. All other data are available from corresponding authors upon reasonable request.

## Supplementary Materials

The following supporting information can be downloaded at: https://www.mdpi.com/article/10.3390/molecules28020805/s1, Figure S1: Linkage of genes and Gene Ontology Terms. Figure S2: Linkage of Genes and significant KEGG pathways. Table S1: Loading protein quantities for proteomics by protein concentration assay. Table S2: Gene pool for Gene Set Enrichment Analysis. Table S3: Density analysis results of western blotting. Table S4: Significant KEGG pathways and related genes involved in oridonin treatment.
