# Supplementary material for: Oridonin Induces Apoptosis in Esophageal Squamous Cell Carcinoma by Inhibiting Cytoskeletal Protein LASP1 and PDLIM1
Source: Molecules. 2023 Jan 13;28(2):805. doi: 10.3390/molecules28020805 (PMC9862004; doi:10.3390/molecules28020805)
Supplement: Supplementary file 1 [file molecules-28-00805-s001.zip › molecules-2136936-supplementary.pdf]

Oridonin Induces Apoptosis in Esophageal Squamous Cell Carcinoma by Inhibiting Cytoskeletal Protein LASP1 and PDLIM1

Xiaojun Zhang [1,2], Mengtao Xing [1], Yangcheng Ma [1], Zhuangli Zhang [2], Cuipeng Qiu [1], Xiao Wang [1], Zhihong Zhao [2], Zhenyu Ji [2,*] and Jian-Ying Zhang [1,*]

Supplemental Experiment Procedure

Protein Concentration Assay for Gel Loading Proteins

Diluted 2 mg/ml bovine serum albumin (BSA) standard (Bio-Rad, Cat# 500-0002, CA) with 2-DE rehydration buffer (Bio-Rad, Cat# 1632106, CA) to final concentrations of 0.2, 0.4, 0.6, 0.8 and 1.0 mg/ml, while blank was 0 mg/ml. Unknown samples of cell lysates were diluted by the same volume 2-DE rehydration buffer, triplicate. Add 250µl 1×dye reagent to 96-well microplate wells. Add 5µl of each standard and unknown sample into each well and mix them well. Incubate at room temperature for 45 min. Then read the absorbance at 595 nm with a microplate reader. Regression linear line by absorbance of standard samples and calculate the protein concentrations. The R square of the linear line should be >0.99.

Supplemental Data

Table S1: Loading Protein Quantities for Proteomics by Protein Concentration Assay

	OD_{sample} 1	OD_{sample} 2	OD_{sample} 3	$Mean_{OD}$	SEM_{OD} (%)	Protein Concentration ×2 (mg/ml)
Untreated	0.5683	0.6561	0.6885	0.6376	3.6	1.097
Treated	0.5743	0.7061	0.6352	0.6385	3.8	1.100

Table S2: Gene Pool for Gene Set Enrichment Analysis

No.	Gene Name	Protein Description	Ref.
1	UBE2V2	Ubiquitin-conjugating enzyme E2 variant 2	a
2	UBE2V1	Ubiquitin-conjugating enzyme E2 variant 1	a
3	ANXA2	Annexin A2	a
4	VDAC2	Outer mitochondrial membrane protein porin 2	a
5	ENO1	Alpha-enolase	a, [1]
6	PDLIM1	Epididymis secretory protein Li 112	a
7	LASP1	LIM and SH3 domain protein 1	a
8	HNRNPH3	Heterogeneous nuclear ribonucleoprotein H3	a
9	HSPA1A	Hsp70 1A	[1-4]
10	HSPA1B	Hsp70 1B	[1, 2]
11	PRDX1	PRDX1	[1]
12	CFL1	COF1	[1]
13	NCL	Nucleolin	[5]
14	STIP1	Stress-induced-phosphoprotein 1	[2, 3]
15	GART	Trifunctional purine biosynthetic protein adenosine-3	[2]
16	STRAP	Serine–threonine kinase receptor-associated protein	[2]
17	PCBP1 (hnRNP E1)	Poly(rC)-binding protein 1	[2, 6]
18	PPA1	Inorganic pyrophosphatase	[2]
19	CBX1	Chromobox protein homolog 1	[2]
20	TPT1	Translationally-controlled tumor protein	[2]
21	GARS1	Glycyl-tRNA synthetase	[2]
22	STMN1	Stathmin	[4]
23	DHFR	Dihydrofolate reductase	[4]
24	PRDX2	Peroxiredoxin-2	[3]

a. Proteins and genes encoding them identified by this study.

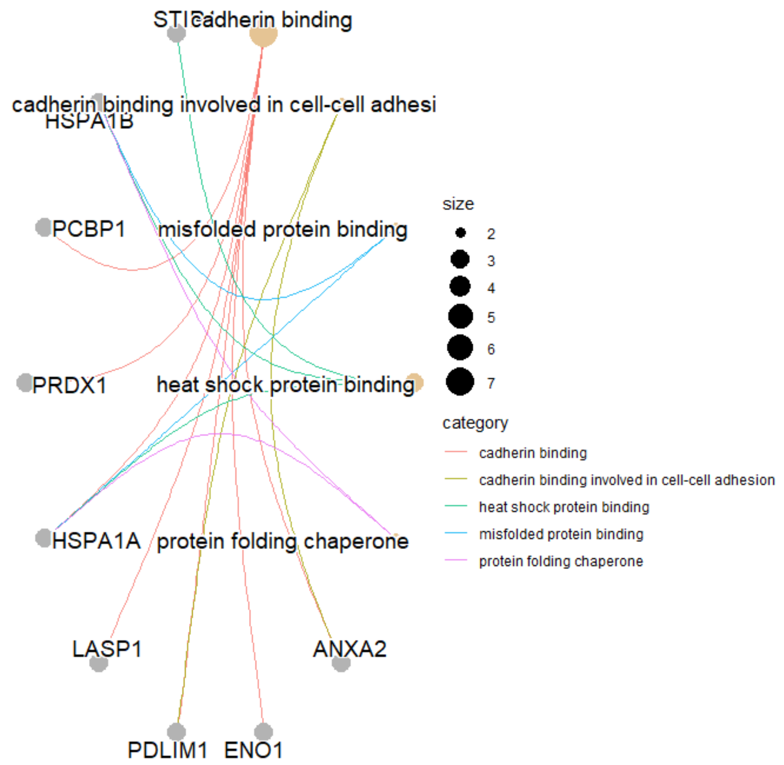

A. The top three significant molecular functions related genes in the pool

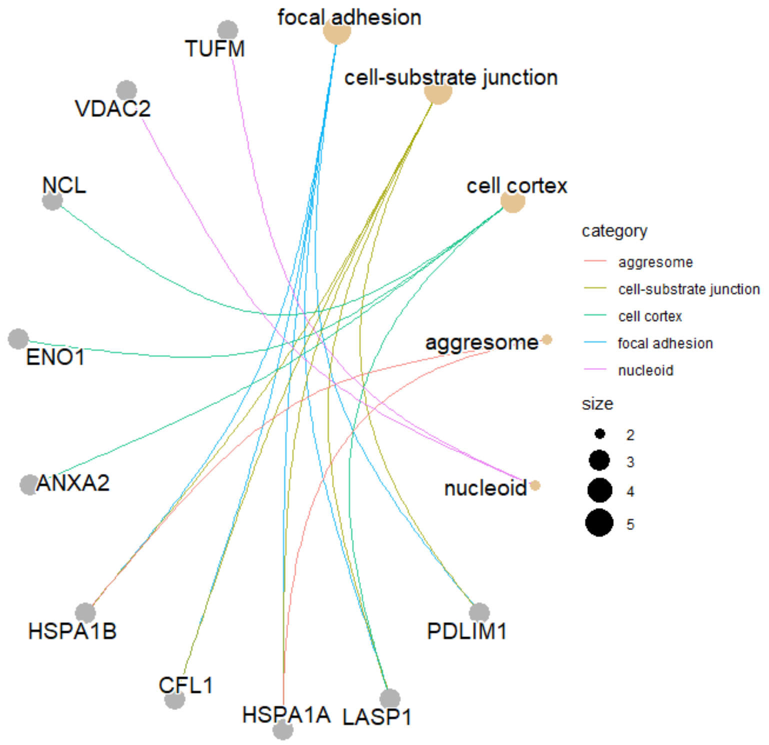

B. The top three significant cellular components related genes in the pool

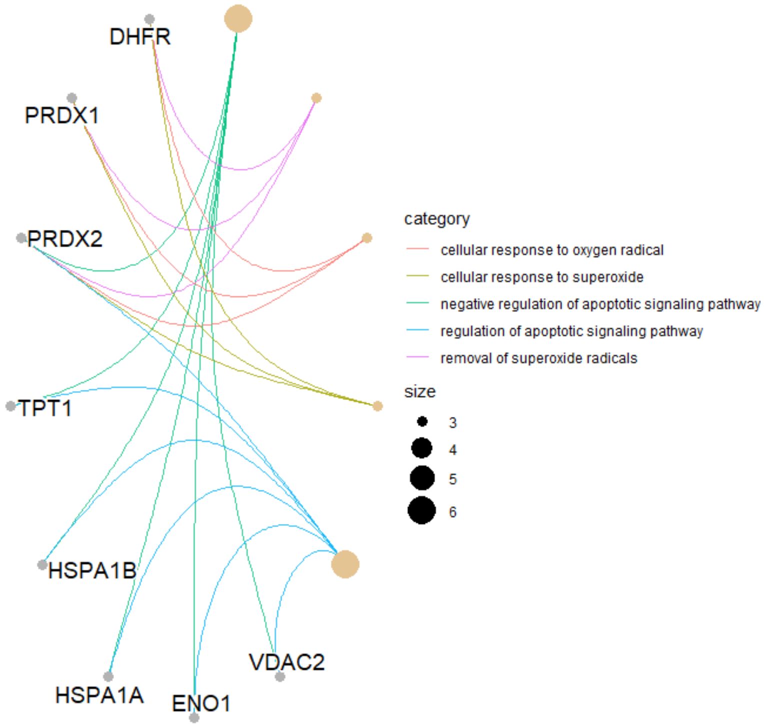

C. The top three significant biological processes related genes in the pool

Figure S1: Linkages of Genes and Gene Ontology Terms. A. The top three significant molecular functions related to genes in the pool. The genes related to cadherin binding are *PCBP1, PRDX1, HSPA1A, LASP1, PDLIM1, ENO1*, and *ANXA2*. The genes related to cadherin binding involved in cell-cell adhesion are *PDLIM1* and *ANXA2*. The genes related to heat shock protein binding are *HAPA1A* and *HAPA1B.* B. The top three significant cellular components related to genes in the pool. Both the focal adhesion and cell-substrate junction-related genes are *HSPA1B, CFL1, HSPA1A, LASP1*, and P*DLIM1*. The cell cortex-related genes are *NCL, ENO1, ANXA2*, and *LASP1*. C. The top three significant biological processes related to genes in the pool. The six genes, *PRDX2, TPT1, HSPA1B, HASP1A, ENO1*, and *VDAC2*, are related to the biological process of negative regulation of apoptotic signaling pathway. The three genes, *DHFR, PRDX1*, and *PRDX2*, are all related to the biological processes of removal of superoxide radicals and cellular response to oxygen radical.

Table S3: Density Analysis Results of Western Blotting

	TE-8		TE-2	
	Untreated	Treated	Untreated	Treated
Hsp70	3043066	3931456	3030770	3383623
GAPDH	2082006	1840684	3132130	2943151
Ratio (Hsp70/GAPDH)	1.46	2.14	0.97	1.15
ENO1	1832870	1191492	2285914	1940519
GAPDH	2306307	1910176	2991397	2356691
Ratio (ENO1/GAPDH)	0.79	0.62	0.76	0.82
LASP1	3737586	177599	3376001	414646
β-Actin	4443588	4225085	5048879	4074327
Ratio (LASP1/β-Actin)	0.84	0.04	0.67	0.10
PDLIM1	5863614	877088	4020970	1506480
β-Actin	5360734	3332648	2734860	2010630
Ratio (PDLIM1/β-Actin)	1.09	0.26	1.47	0.75

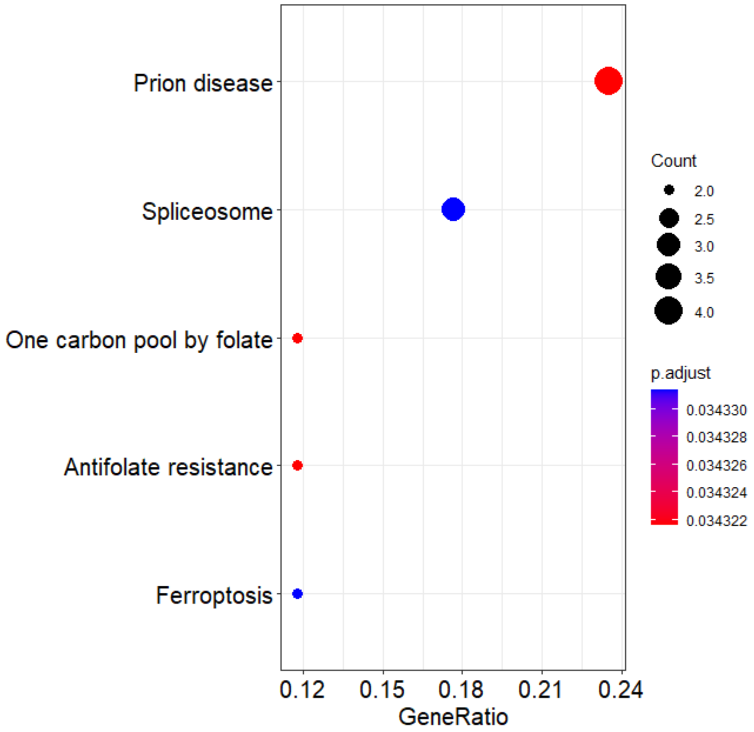

A. Dotplot of the top five significant KEGG pathways

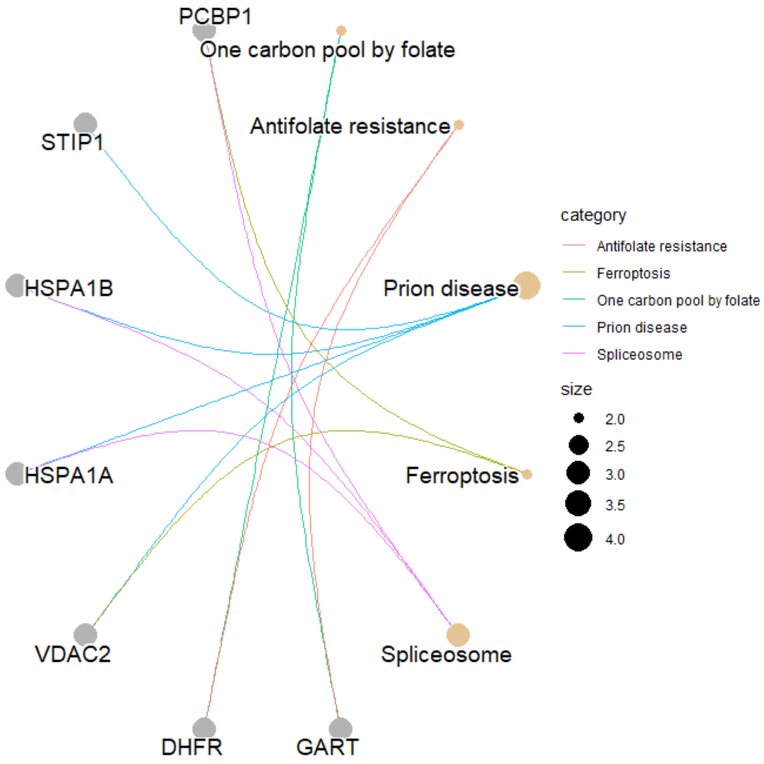

B. The top five significant KEGG pathway related genes in the pool

Figure S2: Linkages of Genes and significant KEGG Pathways. A. The top five significant KEGG pathways are prion disease, spliceosome, one carbon pool by folate, antifolate resistance and ferroptosis. B. The top five significant KEGG pathways related genes in the pool. The four genes, *HASP1A, HSPA1B, VDAC2* and *STIP1* are related to the prion disease. The spliceosome-related genes are *HASP1A, HSPA1B,* and *PCBP1*. Both the one carbon pool by folate and antifolate resistance-related genes are *DHFR* and *GART*. The ferroptosis-related genes are *VDAC2* and *PCBP1*.

Table S4: Significant KEGG Pathways and Related Genes Involved in Oridonin Treatment

	**HSPA1A (2)**	**HSPA1B (2)**	**DHFR (2)**	**GART (2)**	**PCBP1 (2)**	**VDAC2 (2)**	**STIP1 (2)**
Prion disease	X	X				X	X
Spliceosome	X	X			X		
One carbon pool by folate			X	X			
Antifolate resistance			X	X			
Ferroptosis					X	X	

References

1. Dal Piaz, F., R. Cotugno, L. Lepore, A. Vassallo, N. Malafronte, G. Lauro, G. Bifulco, M.A. Belisario, and N. De Tommasi, *Chemical proteomics reveals HSP70 1A as a target for the anticancer diterpene oridonin in Jurkat cells.* Journal of proteomics, 2013. **82**: p. 14-26.
2. Wang, H., Y. Ye, S.-Y. Pan, G.-Y. Zhu, Y.-W. Li, D.W. Fong, and Z.-L. Yu, *Proteomic identification of proteins involved in the anticancer activities of oridonin in HepG2 cells.* Phytomedicine, 2011. **18**(2-3): p. 163-169.
3. Wang, H., Y. Ye, and Z.-L. Yu, *Proteomic and functional analyses demonstrate the involvement of oxidative stress in the anticancer activities of oridonin in HepG2 cells.* Oncology reports, 2014. **31**(5): p. 2165-2172.
4. Zhao, J., M. Zhang, P. He, J. Zhao, Y. Chen, J. Qi, and Y. Wang, *Proteomic analysis of oridonin-induced apoptosis in multiple myeloma cells.* Molecular Medicine Reports, 2017. **15**(4): p. 1807-1815.
5. Vasaturo, M., R. Cotugno, L. Fiengo, C. Vinegoni, F. Dal Piaz, and N. De Tommasi, *The anti-tumor diterpene oridonin is a direct inhibitor of Nucleolin in cancer cells.* Scientific reports, 2018. **8**(1): p. 1-13.
6. Wang, H., Y. Ye, J.-H. Chu, G.-Y. Zhu, W.-F. Fong, and Z.-L. Yu, *Proteomic and functional analyses reveal the potential involvement of endoplasmic reticulum stress and α-CP1 in the anticancer activities of oridonin in HepG2 cells.* Integrative Cancer Therapies, 2011. **10**(2): p. 160-167.
